# Supplementary material for: Are carbon emissions trading and green financial instruments synergistic? -Comprehensive quantitative research based on content analysis
Source: PLoS One. 2024 Mar 7;19(3):e0298601. doi: 10.1371/journal.pone.0298601 (PMC10919601; doi:10.1371/journal.pone.0298601)
Supplement: S1 Data — (ZIP) [file pone.0298601.s001.zip › Data/地级市Z_F工作报告.zip!/░▓╗╒/░▓╟∞╩╨/└┤╫╘╣1⁄2╓┌║┼í╛┬φ┐╦╩2╛▌═°í┐_2011.docx]

政府工作报告

——2011年1月12日在安庆市第十五届人民代表大会第三次会议上
市长  肖超英

各位代表：
现在，我代表市人民政府，向大会报告政府工作，请连同《安庆市国民经济和社会发展第十二个五年规划纲要（草案）》一并审议，并请市政协委员和其他列席人员提出意见。

一、2010年工作及“十一五”发展回顾

刚刚过去的2010年，全市人民在中共安庆市委的坚强领导下，全力抢抓皖江城市带承接产业转移示范区建设的战略机遇，奋力抗击新世纪以来特大的洪涝灾害，全面完成市十五届人大二次会议确定的各项任务。经济社会发展呈现出速度加快、效益提升、民生改善、和谐稳定的良好局面。预计地区生产总值接近一千亿元，增长13.5%；财政一般预算收入121.1亿元，增长25.8%；固定资产投资780亿元，增长30%；社会消费品零售总额340亿元，增长19.2%；进出口总额6.5亿美元，增长30%；城镇居民人均可支配收入14900元，增长10.7%；农民人均纯收入4800元，增长13%；城镇登记失业率控制在4.5%以内，人口自然增长率控制在6.5‰以内。节能减排任务全面完成。
（一）示范区建设全面启动，承接产业转移势头强劲。大力实施示范区规划，开发园区扩容升级步伐加快。安庆经济技术开发区成功晋升国家级。安庆化学工业区加快建设，初步形成4平方公里骨干路网。安庆临港开发区、宿松临江产业园、大观海口工业园获省政府批准筹建。全市省级开发区增加到14个，开发面积达到90平方公里。积极开展与长三角、珠三角地区和海西经济区合作交流，先后赴北京、上海、南京、厦门及皖江地区加强对接，与南京市政府签订融入南京都市圈战略协议。望江与绍兴合作共建纺织服装产业园区。积极参加西洽会、徽商大会、中博会和世博会安徽周等重大经贸洽谈活动，推进开放融合。实际利用市外资金586.62亿元，增长21.34%；利用境外资金2.35亿美元，增长16.3%。
（二）有效投入不断扩大，发展动力进一步增强。扎实开展重点项目突破年活动，加快省“861”和市“3231”等重大项目建设，强力推进42个重大项目实施。安庆石化炼化一体化、林浆纸一体化、宁安城际铁路及安庆长江铁路大桥建设进展顺利，望东长江公路大桥接线、石门湖航道整治等一批项目开工建设。500千伏文都和双岭变电站建成投运。完成重点项目投资248亿元。争取中央和省补助资金11.3亿元。开工与央企合作项目14个。加强同全国知名民营企业合作发展，419个项目成功签约。引进浦发、兴业、中信3家股份制银行。加快发展地方金融机构，新增小额贷款公司8家，3家农信社完成改建农村银行。各项贷款余额556.3亿元，增长23.8%。直接融资工作取得新突破，桐城盛运股份公司在深圳创业板上市。
（三）工业经济提速增效，自主创新积极进展。规模以上工业企业1548户，净增269户。产值超亿元企业超过200户。工业投资380亿元，增长35.7%。华茂30万锭紧密纺、东方造船30万吨一期、中船柴油机基地、怀宁上峰水泥一期、丹凤集团一号池窑、潜山利达汽车轴承等项目建成投产，建成国家石化产品质量监督检验中心。华茂集团转让国有股权，建设华茂国际纺织工业城。非公有制经济发展活力进一步释放，新增私营企业3335户。支持企业技术改造，列入省“百千工程”项目280个，总投资311亿元，列入国家和省技术创新项目67个。推进产学研合作，华茂集团和海军4812厂研制的非晶软磁材料及制带设备取得重大突破。扎实开展国家知识产权试点城市建设，专利申请量562件，增长31.3%。中国驰名商标和中国名牌产品分别达到6个。
（四）“三农”工作持续加强，城乡统筹成效明显。农业生产喜获丰收，粮食总产达268万吨。土地流转面积126万亩。狠抓“菜篮子”工程，建成市郊蔬菜基地2万亩。油茶等经果林基地建设加快。枞阳、太湖启动农村土地整治示范建设。全面落实各项强农惠农政策，发放各类涉农补贴6亿多元。花凉亭等38座大中小型水库除险加固工程完工。下浒山水库建设项目获国家批准。建成安全饮水工程214处，解决25.6万农村人口饮水安全问题。新建农村户用沼气16950户，修建村村通公路775公里。加强扶贫工作，实施整村推进125个村，减少绝对贫困人口7万人。建立村级公益事业建设“一事一议”财政奖补机制，开展惠民直达工程试点。阳光村务工程有效推进。
（五）旅游经济蓬勃发展，服务业加快提升。天柱山创建国家5A景区，菱湖风景区、独秀园、五千年文博园、嬉子湖创建4A景区通过国家旅游局验收。文化旅游加速融合，潜山山谷流泉摩崖石刻文化园、岳西映山红大观园等景区建成。桐城孔城老街、岳西温泉、宿松石莲洞、枞阳浮山等项目加快建设。举办中国水上旅游论坛，上海世博旅游专列开行安庆，豪华游轮常态化停靠。天柱山机场旅客吞吐量近8万人，创历史新高。旅游总收入127.6亿元，增长30.1%。做好家电、汽车摩托车下乡和以旧换新工作,销售额30亿元。完善城乡市场体系，“新网工程”逐步规范。舒怡、神墩家居广场建成开业，远航港口物流园、粮食物流园加快建设。房地产市场平稳健康发展，房地产交易额79.83亿元，增长31%。
（六）宜城崛起积极推进，城市建设步伐加快。以创建省级文明城市为契机，开展宜城崛起推进年和城市大建设活动。新开工项目40个，续建项目43个，城建十大重点工程扎实推进。机场大道、元山路二期、顺安路二期、马山东路工程基本完工，振风大道二期、华中东路三期加快推进。实施集贤关和花亭大沟区域综合整治工程，启动东部水系综合治理，城市排污排水应急能力增强。城市燃气管网新改建工程基本完工，城区天然气置换4万余户。东部新城综合写字楼启动建设，市体育中心完成规划选址，北部新城、大观新城建设加速。实施依法征迁拆违和建设环境专项整治，市区共拆迁100万平方米，集中拆除违法建设5万平方米，实现和谐拆迁。
（七）节能减排扎实开展，环境保护取得实效。开展千家企业节能行动和节能产品惠民工程。加强对47户高耗能企业监管。强化重点行业节能降耗管理，大力推进建筑、交通运输、公共机构和农村等领域的节能。严格项目节能评估，坚决控制高能耗项目进入，切实淘汰落后生产能力。大观循环经济产业园发展加快。国家级区域性大型再生资源回收利用基地获批。切实解决群众关切的大气恶臭和水体污染问题。严肃查处破坏环境违法违规行为，实施工业园区和重点行业环保专项行动。马窝污水处理厂主厂区建成,八县（市）污水处理厂正常运营。
（八）民生工程深入实施，社会保障更加有力。省定33项、市定9项民生工程顺利推进，投入资金39.84亿元。稳定和扩大就业，城镇新增就业5.51万人，转移农村劳动力就业5.5万人，帮扶4297名困难人员实现就业。不断完善社会保障体系，基本养老、基本医疗、失业保险参保人数分别为33.2万人、39.1万人、25.8万人。全面实施工伤保险、居民医疗保险市级统筹和养老保险基金市级统筹统支，20.2万名被征地农民应保尽保，其中40267名达龄被征地农民按月领取养老保障金。新农保试点在怀宁、潜山有序展开。新建、改扩建农村敬老院83所，农村五保户供养标准和集中供养率进一步提高。加快推进保障性安居工程，新增廉租住房8403套，为4673户职工购房提供住房公积金支持。完成11条小街小巷改造、2463盏街巷路灯安装，深受老百姓欢迎。关注弱势群体救助，7.3万人享受低保，4.4万人享受贫困重度残疾人生活救助。及时启动市场价格监督应急预案，有效控制物价上涨对城乡居民生活的影响。
（九）社会事业加快发展，公共服务水平提升。安庆师范学院加快建设和发展，安庆职业技术学院获准建设省示范高职院校，桐城师范升为大专。中小学校舍安全工程全面实施。大力实施文化惠民工程，建成乡镇综合文化站56个，农家书屋313个。加强文化交流和市场开拓，组织开展公益演出近百场，再芬剧院成功赴美访演。文化体制改革重点任务完成，文艺院团改革基本完成。市博物馆工程启动建设。深入推进基层医疗卫生体制综合改革，实现基本药物零差率销售，基本药物价格平均下降40%。参加第十二届省运会夺得金牌74枚，成功获得第十三届省运会和第六届省残运会举办权。建成146个村级农民体育健身工程。计划生育保障体系逐步完善，低生育水平保持稳定。畅通信访渠道，开展维稳综治专项活动，人民群众安全感明显提高。安全生产形势总体平稳。产品质量和食品药品监管力度加大。认真开展第六次全国人口普查。国防动员、人民防空、国家安全和双拥工作扎实推进。民族宗教、外事侨务、妇女、儿童、老年人、残疾人等各项事业实现新发展。
（十）始终坚持依法行政，自身建设不断加强。坚持市政府常务会议学法制度，加快推进政府职能转变，法治环境进一步优化。“五五”普法顺利验收。自觉接受人大法律监督和政协民主监督，办理人大代表议案和建议153件、政协提案415件，办复率100%,满意率不断提高。进一步密切与工会、共青团、妇联等人民团体的联系。切实加强政务公开，加快电子政务建设。重视开展网络问政，认真办理网民留言回复。凝聚各方智慧，完成“十二五”规划编制。落实党风廉政建设责任制，严肃查处违法违纪案件。 
各位代表！2010年是“十一五”收官之年，全市经济社会保持平稳较快发展，确保了“十一五”规划的圆满完成。过去的五年，是安庆发展史上极不平凡的五年。
这五年，经济实力不断增强。地区生产总值年均增长12.4%，高于“十五”平均增速3.4个百分点，千亿安庆目标基本实现。财政一般预算收入突破百亿大关，增长2.2倍。工业总产值达到1350亿元，增长2.8倍。金融机构存款余额达1136.4亿元，增长1.7倍。
这五年，双百城市框架基本形成。全市城镇化率达42%，比“十五”末提高9个百分点。中心城市加速拓展，东进北扩西拓进程加快,基本形成建成区面积100平方公里框架。成功创建国家园林城市，皖江公园、江滩公园建成开放，菱湖风景区环境综合治理工程获国家人居环境范例奖。
这五年，经济发展协调性明显增强。实现骨干企业翻番和园区经济倍增“两个计划”，规模以上工业增加值达到360亿元，规模以上工业企业净增1056家。高新技术产业产值由5亿元增加到95亿元。旅游总收入年均增长20%以上，五年累计400亿元，是“十五”总量的2.56倍，成为全省第二大旅游经济区。三次产业比重由“十五”末的21.2:40.4:38.4调整为16.5:51:32.5。
这五年，发展基础不断夯实。固定资产投资累计完成2389亿元，是“十五”时期的5倍。谋划并建成了一批打基础、管长远、增后劲的大项目,累计竣工重点项目440个，完成投资800亿元。建成公路总里程1.5万公里，其中村村通水泥（油）路6900多公里。水利基础设施五年投入31.2亿元，是“十五”时期的1.64倍。电网投资50亿元，新建110千伏及以上变电站14座，全社会用电量比“十五”末增长70%。
这五年，发展活力进一步迸发。新增私营企业10038户、个体工商户13238户。金融体制改革取得重大进展，5家农村信用社完成改革。投融资体制改革扎实推进，政府投融资功能大幅提升，累计融集城市建设资金57亿元。对外开放水平不断提升，成功举办两届黄梅戏艺术节。五年累计引进市外资金1888亿元，年均增长42.36%。实际利用境外资金8.68亿美元，实现进出口总额22.7亿美元，分别是“十五”时期的2.5倍和2.66倍。
这五年，人民生活极大改善。民生工程累计投入财政资金93.79亿元。就业规模持续扩大，城镇新增就业22.5万人，下岗失业人员再就业11.6万人，就业困难人员再就业2.1万人。136.9万农村人口饮水安全问题得到解决。2010年城镇居民人均可支配收入、农民人均纯收入比“十五”末分别增长77.5%和83.8%。开发式扶贫成效明显，投入各类扶贫资金11.73亿元，五个扶贫重点县经济总量翻了一番，减少绝对贫困人口54万人。
各位代表！五年的成就来之不易，这是省委、省政府和市委正确领导的结果，是全市人民团结拼搏、开拓进取的结果！在此，我谨代表市人民政府，向全市广大工人、农民、知识分子、干部、驻宜解放军指战员、武警、消防官兵和政法干警，向各民主党派、无党派、各人民团体和社会各界人士，致以崇高的敬意！向关心支持安庆发展的中央和省驻宜单位，向在宜的所有投资者和建设者，表示诚挚的感谢！
在肯定成绩的同时，我们也清醒地认识到,我市经济社会发展中还存在不少困难和问题。主要是：人均发展水平在全省靠后，经济持续稳定增长的基础还不稳固；县域经济发展不平衡，中心城市辐射带动力不强，统筹发展任重道远；经济结构调整压力加大，自主创新水平不高；资源环境约束强化，节能减排任务艰巨；土地征用、房屋拆迁等方面还存在一些群众关切的问题；机关作风和效能建设需要进一步加强。对此,我们一定高度重视，认真加以解决。

二、奋力谱写“十二五”发展新篇章

“十二五”时期是安庆全面建设小康社会的关键期，是大有可为的黄金发展期。国家宏观政策环境总体有利，承接产业转移机遇前所未有，工业化城镇化加速推进，长期大规模投入效应加速释放，这些都是支撑“十二五”发展的积极因素。只要我们牢牢把握机遇，用好机遇，就一定能够实现经济平稳较快发展，推动安庆科学发展、全面转型、加速崛起。站在新的历史起点，我们必须进一步增强责任意识，更加坚定加快发展的信心和决心,不断开创安庆现代化建设新局面。
根据市委九届十三次全会通过的《关于制定国民经济和社会发展第十二个五年规划的建议》，“十二五”时期的发展，必须以邓小平理论和“三个代表”重要思想为指导，深入贯彻落实科学发展观，以科学发展为主题，以全面转型、加速崛起、富民强市为主线，坚持工业化城镇化“双轮驱动”，大力实施工业强市、三产兴市、创新推动、园区带动、城乡统筹、和谐发展战略，推动经济社会又好又快发展，争取走在皖江发展前列，为实现经济繁荣、人民富足、生态良好的发展目标，全面建成小康社会奠定坚实基础。
在市委《建议》指导下，市政府编制了《十二五规划纲要（草案）》。综合考虑，今后五年发展的主要预期目标是：力争“十二五”期间主要经济指标增幅超过全省平均水平，到2015年人均生产总值达到全省平均水平；确保地区生产总值、财政收入、工业增加值、服务业增加值比2010年翻一番，力争城乡居民收入翻一番。
实现“十二五”发展目标，必须重点把握以下五个方面：
（一）坚持加快发展。深入实施“3231”行动计划，推动工业化和城镇化协调共进，强化园区带动力，是建设区域中心城市的重要引擎，必将为加快发展释放巨大的能量。
加快新型工业化进程。优化产业空间布局，着力构建“一核两带两轴六组团”产业框架。壮大提升优势产业，努力打造石油化工、纺织服装、机械和装备制造三大千亿产业，基本建成全国重要的石化和轻纺产业基地。力争在新材料、新能源、电子信息、生物医药、节能环保等方面取得突破。全面提升信息化水平。持续增强主导产业实力，大幅提高战略性新兴产业对经济增长的贡献率。
加快新型城镇化进程。加快以中心城市为核心，以八县（市）县城为依托的安庆城市群建设，打造带动皖西南、辐射皖鄂赣交界地区的区域中心城市。加快东部新城、北部新城和大观新城配套建设，建成政务中心、商务中心、体育中心和金融中心。扩大宜城板块，形成“大宜城”框架。加速推进历史文化和现代文明相互融合，打造现代化历史文化名城。积极创建国家森林城市，建设山水宜居宜游城市。加强城乡规划管理，高标准建设各类市政设施，提高城市基础设施承载能力。稳步推进房地产业健康发展。构建以中心城市为龙头、县城、重点镇和村庄为支撑的现代城镇体系。
强化园区带动力。以园区为载体，带动工业集聚、城镇发展。引导资源、要素和项目向园区集中，确保园区经济年均增速40%以上。加快园区扩容升级，确保到“十二五”末，园区开发面积翻一番，生产总值达到全市一半。努力实现国家级开发区综合实力大幅提升，积极申报国家出口加工区。加强特色园区建设，形成一批产业园区、生态工业园区和循环经济园区，实现产业错位发展。努力将化学工业区打造成千亿园区。
（二）坚持转型发展。经济结构调整优化是转型发展的主攻方向，必须努力实现需求结构与产业结构优化，资源节约与环境友好并重，实现全面协调可持续发展。
推动三大需求协同增长。坚持扩大内需战略，持续做好项目工作，扩大有效投入。推进阜景铁路安庆段、合安城际铁路、合九铁路复线、安庆长江公路二桥建设。推进岳武高速、北沿江高速、老洲-宜城-望江-宿松-九江快速通道建设。加快天柱山机场改造，规划安庆新机场。建成石化炼化一体化、林浆纸一体化、宁安城际铁路安庆段及安庆长江铁路大桥、望东长江公路大桥、长风港一期工程，完成石门湖航道整治，加快推进安庆核电项目。推动智能电网建设，新建变电站30座。促进消费结构升级，努力扩大消费需求。大力开拓国际市场，扩大出口规模，优化出口结构。强化出口产业集群和出口基地建设，增强出口产品市场竞争力。
切实优化产业结构。构建以现代农业为基础、战略性新兴产业为先导、先进制造业和现代服务业为支撑的格局,实现经济增长由主要依靠第二产业带动向依靠三次产业协同带动转变。把促进服务业大发展作为产业结构调整的战略重点，大力发展现代服务业。加快发展以黄梅戏为主体的演艺娱乐业，以山水人文资源为依托的文化旅游业，以体现地域文化为特色的文化创意业，打造文化品牌，做大做强文化产业。加快大别山旅游区建设，努力把安庆打造成泛长三角和中部地区重要的文化旅游休闲基地、国内一流的旅游目的地。加快发展商贸物流业，构建区域商贸物流中心。
加快构建两型社会。围绕建设资源节约型和环境友好型社会，强化节能减排目标责任制，提高生态环境质量。加强污染防治和环境保护，有效控制和削减二氧化硫排放总量，全市空气质量优良率达到90%以上。落实化学需氧量排放量、氨氮和氮氧化物减排目标，确保污染源达标排放。加强资源节约集约利用，抓好化工、纺织、建材、建筑等重点行业节能。大力发展循环经济和低碳经济。
（三）坚持创新发展。创新是科学发展、全面转型的持久动力。大力实施创新推动战略，深入推进重点领域和关键环节改革，创新体制机制，促进开放发展。
加快推进自主创新。积极参与国家技术创新工程试点省建设，着力构建企业主体、市场导向、政府推动、产学研用结合的开放型创新体系。支持各类企业建设研发机构。推动企业与高等院校、科研机构的科技合作与共建，构筑科技资源共享平台。全面加强知识产权示范创建市建设。引进培养高层次、创新型、复合型和技能实用型人才，强化智力支撑。
着力完善体制机制。稳妥推进事业单位、财税体制、收入分配制度和非基本公共服务市场化改革。加强以产权多元化为核心的企业改革。探索金融支持示范区建设的新途径，加快股份制银行引进和小额贷款公司等新型金融组织组建。积极培育企业上市。加快发展非公有制经济。推进农村各项改革试点，逐步建立城乡统筹的土地利用制度和建设用地市场。全面推进集体林权制度配套改革。适时启动行政区划调整。
积极承接产业转移。以示范区建设为动力，围绕产业结构升级和新兴产业培育，推进产业集群式招商。加强与沿海发达地区以及大企业、大集团和战略投资者的合作，推进组团式承接产业转移，提升引资质量和效益。积极参与泛长三角等区域分工合作，着力在体制机制及基础设施对接、市场融合、旅游合作、劳务输出等方面开展交流合作。加强区域协作，促进融合发展。
（四）坚持统筹发展。作为农业大市，必须坚持把解决好“三农”问题作为全市工作的重中之重，建设农民幸福生活的美好家园。
加快发展县域经济。统筹推进县域工业化、城镇化和新农村建设，加快农业产业化、农村社区化、农民现代化进程。进一步做大做强县域经济支柱产业，着力形成优势产业带和区域城镇群，实现规模与效益的快速提升。深化扩权强县和扩权强镇，激发县域活力，切实提高县域经济在全市经济发展中的比重。
完善现代农业产业体系。围绕优质粮、棉、油、茶、畜禽、水产六大主导产业，大力推进农业产业化，国家和省级龙头企业达到100家以上。加大科技投入，积极发展绿色生态农业和观光休闲农业。完善农村社会化服务体系，加快发展农民专业合作组织。着力推进农业规模化、集约化、机械化、标准化生产，不断提高农业综合生产能力。
改善农村生产生活条件。依法推进土地流转，发展适度规模经营。加强农村道路、电网、通信、排水、垃圾处理和高标准农田等基础设施建设。完善水利保障体系，提升对自然灾害的综合防御能力。加强饮用水源保护，继续实施农村饮水安全工程。规划实施华阳河分蓄洪区安全工程，建成下浒山水库。
有效提高农民收入。提高农民职业技能和创收能力，多渠道增加农民收入。鼓励农民优化种养结构，提高效益，增加农民生产经营收入。加快发展农产品加工业和非农产业，促进农民转移就业，增加工资性收入。认真落实各项强农惠农政策。以促进贫困农民脱贫致富为核心，全面推进新时期扶贫开发。加大对贫困地区支持力度，实施产业化扶贫和整村推进工程。
（五）坚持和谐发展。顺应人民群众的新期待、新需求，必须加大对社会建设的投入，持续实施民生工程，逐步完善覆盖城乡、可持续的基本公共服务体系。
构建覆盖城乡的就业和社会保障体系。实施更加积极的就业政策，建立健全就业帮扶制度和促进就业长效机制，千方百计扩大就业。实现新型农村社会养老保险全覆盖，完善城镇职工养老保险制度，积极推进城镇居民养老保险制度建设。稳步推进机关事业单位养老保险制度改革。探索城镇居民医疗保险制度改革。扩大社会保障覆盖范围，不断提高保障标准。加快社区养老服务机构建设。加强社会福利事业，实现城乡社会救助全覆盖。
统筹发展社会事业。推动文化大发展大繁荣。深化文化体制改革。系统研究整理戏曲文化、桐城派文化、皖江文化和禅宗文化。办好中国黄梅戏艺术节。建成市博物馆，提升市县两级综合档案馆水平。实施文化惠民工程，丰富城乡群众文化生活。加快推进广播电视数字化建设，实现广播电视村村通。认真开展教育统筹综合改革试点，推进学前教育、义务教育、职业教育、高等教育协调发展，提升基础教育。深化医药卫生体制改革，加强公共卫生服务体系建设。积极发展体育事业，建成市体育中心等一批体育场馆，精心举办第十三届省运会和第六届省残运会。
加强和创新社会管理。加强民主法制建设，全面推进依法治市，构筑促进和谐的社会基础。加强国防动员建设，切实做好双拥工作。做好人口与计划生育工作，保持人口均衡发展。积极应对人口老龄化，切实保护妇女儿童合法权益，支持残疾人事业发展。完善突发事件应急管理机制，建立健全社会预警体系，提高保障公共安全和处置各类突发事件的能力。创新社会管理方式，加强网络社会监管，加强社会治安数字化防控体系规划建设，严厉打击违法犯罪。落实安全生产、社会治安和维护稳定的各项措施，努力形成社会安定、百姓安宁的良好局面。

三、着力抓好2011年各项工作

做好今年工作，实现“十二五”发展良好开局，对于推动我市加快发展、全面转型、富民强市，具有十分重要的意义。
综合考虑，今年全市经济社会发展的主要预期目标是：地区生产总值增长13%以上，财政一般预算收入增长17%，固定资产投资增长25%，社会消费品零售总额增长19%，进出口总额增长25%，城镇居民人均可支配收入增长12%，农民人均纯收入增长13%，居民消费价格指数4%左右，城镇登记失业率控制在4.5%以内，人口自然增长率、节能减排等约束性指标完成省下达任务。
实现上述目标，必须着力抓好以下八个方面工作：
（一）贯彻宏观调控政策，保持经济平稳较快增长
认真落实国家宏观调控政策，切实增投入、扩内需、稳外需，加强经济运行调节，增强经济发展动力，继续稳定价格总水平，努力保持经济平稳较快增长。
坚持不懈扩大有效需求。扎实推进省“861”和市“3231”行动计划，强化项目责任制，提高投资质量和效益，确保固定资产投资稳定增长。力争开工建设望东长江大桥、岳武高速、合安城际铁路、热电联供、宿松风力发电一期等工程。抓好北沿江高速、桐城至池州高速等项目前期工作，争取早日开工建设。大力开拓城乡市场，积极引导消费结构升级，着力提高城乡居民消费能力。深入实施“万村千乡市场工程”、“社区双进工程”和“新网工程”，继续推进家电、农机、建材下乡活动。积极发展物流配送、连锁经营、电子商务等新型业态，培育发展文体健身等消费热点，支持和规范网上消费。加强房地产信息化建设和市场监管。积极支持外贸企业做大做强，推动加工贸易转型升级。巩固纺织、机电、农产品等出口，扩大高新技术和高附加值产品出口份额，促进外贸出口增长。加强口岸建设，扩大本地进出口规模。
加快园区经济发展。进一步完善园区发展规划，优化产业空间布局，促进园区转型升级。新增开发面积30平方公里，力争园区基础设施建设投资和工业总产值增长40%以上。培育一批百亿园区。加速安庆临港、桐城双新、宿松临江、望江桥港等开发园区建设，推进怀宁茶岭、枞阳横埠、潜山源潭等一批特色园区申报筹建，扩大园区规模，增强承接能力。支持安庆经济技术开发区发展提速增效，加快拓展三期。建设台资工业园。加速化学工业区基础设施建设和项目进区入园，积极筹建省级高新技术开发区，争创国家高新技术开发区。用好用活先行先试权，推进园区整合，重点建设10个合作园区。鼓励社会资本参与建设、经营园区基础设施和公用事业，增强园区发展活力。
持续强力招商引资。围绕示范区产业发展指导目录和园区定位，全面落实各项优惠政策，充分利用徽商大会、中博会等重要平台，大力招商引资，确保全市利用市外资金增长20%以上。继续坚持市直招商，加强驻外招商。突出招大引强，推进与央企、全国知名民企合作，重点承接和引进一批产业层次高、投资规模大、带动能力强的产业和项目。推进专题招商和产业招商，认真举办汽车零部件专题招商、石油化工和临港产业招商、新能源项目招商等活动。跟踪落实已签约项目，积极调度亿元以上重大招商项目，提高合同履约率、资金到位率、项目开工率和投入产出率。坚持绿色承接，加强环境监测，严把产业准入门槛。
强化物价调控监管。围绕影响人民群众切身利益的价格热点难点问题，加强价格监测预警，积极引导社会预期，增强物价调控的针对性，全力保持价格总水平基本稳定。认真抓好生活必需品市场供应，健全储备和应急调控体系。大力开展价格监管，重点加强对农产品市场交易和价格行为的监管，严厉打击恶意炒作等违法行为，整顿和规范市场价格秩序。强化价格调控的社会保障机制，落实各类物价补贴，保障城乡困难群众基本生活。
（二）加快经济结构调整，提升产业核心竞争力
坚持规模扩张和转型升级并重，着力壮大提升优势产业，积极培育战略性新兴产业，加快发展现代服务业，强化节能减排刚性约束功能，全面提升经济发展水平和综合竞争力。
着力壮大优势产业。围绕建设石油化工、纺织服装、机械和装备制造三大千亿产业，做大做强优势产业。加快石化炼化一体化、华茂国际纺织工业城、东方造船基地、安踏工业园、中建材超白压延玻璃生产线、精科大马力拖拉机以及曙光化工集团升级改造、液化气深加工等重大项目建设，发展国家级汽车零部件生产基地。大力发展石油深加工、精细化工，延伸石化产业链。着力提高产业集中度，加快形成纺织服装产业群。推动曙光集团、环新集团、船用柴油机厂、华泰纸业等一批骨干企业提升规模、转型升级，增强核心竞争力。支持非公经济大发展快发展。
大力发展战略性新兴产业。出台扶持政策，建立战略性新兴产业引导基金，着力培育新材料、新能源、节能环保、电子信息、生物医药、高端装备制造业等新兴产业，加快形成产业竞争优势。加快新兴产业项目支撑、科技创新、融资服务“三大平台”建设，为战略性新兴产业发展创造良好条件。大力支持恒昌、鸿庆、盛运等企业发展壮大，推进飞凯高分子材料、光大光伏发电、和兴生物塑料、卫康医药、金张科技等加快发展，加快建设若干专业化程度较高的新兴产业园区。开工建设非晶软磁及复合材料、太阳能光伏玻璃生产线项目。引导发展软件服务业，推动电子信息产品制造上规模上档次。壮大一批“专精特新”企业。
加快发展服务业。深入落实服务业市场准入、税收优惠和用地保障等政策措施，大力发展科技、物流、信息等生产性服务业，加快发展商贸服务、社区服务等生活性服务业，积极发展服务外包等新兴服务业。切实做好服务业综合改革试点，创建全省商贸服务业发展示范区。加快各类物流园区、配送中心和专业市场建设。打造以光彩大市场为龙头，以生活消费品、生产资料为主体的市场集聚区，强化市场的辐射功能。加快实施县区超市建设工程。支持连锁超市、农产品流通企业与农产品专业合作社对接。
扎实抓好节能减排。开展工业节能专项行动，降低工业企业能源和原材料消耗水平。加快淘汰落后生产能力，坚决控制“两高”和产能过剩行业新上项目。加强重点用能企业和建筑、交通运输、公共机构、农村等重点领域和行业的节能降耗。严格实施污染治理处罚和问责措施，促进企业治污设施正常运行。突出工程建设领域环境整治，加强建设项目环境影响评价和“三同时”管理。建成垃圾焚烧发电厂和医疗废弃物集中处置中心，加快马窝污水处理厂及配套设施建设，开工建设北部新城污水处理厂。加强八县（市）污水处理厂正常运营的监管。
（三）加大强农惠农力度，持续夯实“三农”基础
牢固树立强农惠农意识，始终把“三农”工作抓紧抓牢，不断夯实农业农村发展基础。
稳定农产品生产和市场供应。认真落实“菜篮子”市长负责制，抓好农产品生产和流通，稳定2万亩、新建4000亩市郊蔬菜基地。加强农技推广体系建设，推广良种良法，培育一批现代农业科技示范园区。全面实施新增粮食生产能力规划，提升农业综合生产能力。着力培育一批农产品加工龙头企业，积极打造农业特色板块和产业集群。推进皖江农产品生产物流中心建设。稳定粮食播种面积，加快粮食物流园区一期工程建设。实施茶产业振兴、蔬菜产业提升工程。
狠抓农田水利等基础设施建设。推进江河堤防除险、病险水库加固、大型排涝泵站更新改造工程，续建大中型灌区节水改造项目。开工建设下浒山水库，实施大沙河等小流域治理和病险水库闸站除险加固，启动华阳河流域综合治理生态恢复工程。继续实施农村饮水安全、乡村清洁工程。加强农村教育、卫生、文化、电网、社区服务中心等设施建设，建设农村新型社区。新建农村客运站18座。
多渠道促进农民增收。全面落实农业补贴政策，认真执行水稻、小麦、油菜籽等最低收购价，保护和调动农民生产积极性。加强农民培训和农村实用人才开发，千方百计增加农民经营性收入、工资性收入和财产性收入。加快发展高效特色农业、休闲观光农业，挖掘农业内部增收潜力。扩大种苗花卉规模，增加油茶、中药材、经果林基地面积，大力发展林业经济，帮助农民兴林致富。实施畜牧业升级计划和水产跨越工程。建立财政扶贫投入机制，扎实推进开发式扶贫。
进一步深化农村改革。继续深化农村综合改革，健全“一事一议”财政奖补机制。扎实做好集体林权制度配套改革工作，高水平完成农业技术推广、动植物疫病防控、农产品质量监管等公共服务机构改革任务。大力推动农村土地整治示范建设，抓好枞阳、太湖试点工作，发挥示范带动效应。发展农业适度规模经营。编制新增省级新农村建设示范村村庄布点规划和建设规划。建立农村公益设施建设与管护长效机制，探索完善有利于农民进城的就业、住房、户籍改革和社会保障制度，逐步推进城乡之间基本公共服务和社会保障均等化。
（四）加快城市化步伐，构建现代城镇体系
坚持把城镇化作为扩大内需和调整结构的战略重点，形成基本公共服务和基础设施一体化、网络化发展的城镇化新格局。
加快建设皖西南中心城市。以政务中心、商务中心、体育中心、金融中心建设为重点，全面推进城市大建设，完成基础设施投资70亿元以上。开工建设潜江路、柘山路二期、晴岚路南段、顺安路三期、沿江东路中段、环湖西路等道路，改建双岗路、湖心路、曙光路，贯通沿江东路西段，完成机场大道、振风大道二期，续建勇进路、皇冠路、独秀大道北段和环城西路。加快城市排污排水应急工程建设，完成花亭大沟区域综合整治一期工程，启动花亭南北村内涝治理和城东污水处理厂二期工程。实施新河、顺安河整治。开工建设菱湖风景区东侧城市综合体项目。扎实推进老城区改造开发，完成人民路等一批街巷改造。加强历史文化名城保护，修复江西会馆、英王府等。全力推进国家森林城市建设，加强集贤关等重点区域环境综合整治。继续开展依法征迁拆违和建设环境专项整治。高度重视还建房建设，加快速度，提升品质。
加快县城和重点镇发展。加强分类指导，以县城和重点城镇为主体，改善城镇基础设施，完善城镇服务功能。大力发展中心镇，加快建设一批特色经济强镇。继续开展文明县城创建和小城镇建设“宜城杯”竞赛活动。推进小城镇道路、供水排水、园林绿化、电力通信、垃圾处理等基础设施和居住区、产业园区建设，增强城镇吸纳人口、增加就业和聚集产业能力。加快推动城市公共资源向农村倾斜，公共设施向农村延伸。
加强城镇规划建设和管理。坚持规划引领，科学编制城镇规划，合理确定功能布局和产业定位，统筹安排基础设施和公共服务。高标准建设地下管网、综合交通、信息网络、生态环境、防灾减灾等基础设施体系，提高发展承载力。强化城镇建设和管理，开展违法建设和村容镇貌整治。创新城镇管理模式，提升城镇品位，营造整洁有序、和谐舒适、宜居宜业的城镇环境。
（五）深化重点领域改革，增强创新能力
加快重点领域、关键环节改革创新，完善有利于科学发展的体制机制，不断为经济社会发展注入新的动力和活力。
推进重点领域改革。继续深化行政管理体制改革，推进事业单位改革，进一步减少和规范行政审批，切实缩短办事时限，提高办事效率。继续深化财政管理体制改革，加快建立和完善公共财政预算、国有资本经营预算、政府性基金预算和社会保障预算体系。深化基层医药卫生体制综合改革，加快建设覆盖城乡居民的基本医疗卫生服务体系。积极稳妥地推进分配制度改革，加快文化体制等社会事业领域改革，努力形成利于公益、服务社会、推动发展的繁荣局面。
创新投融资方式。强化金融支持，引导金融机构把信贷资金更多地投向“三农”、中小企业和战略性新兴产业。做好政府性融资平台清理和规范工作，提高城投公司、发投公司融资能力和资本运作水平。积极争取信贷额度，力争信贷增幅高于全省平均水平。鼓励金融机构创新金融产品和服务，满足市场需求。加强地方金融机构建设，做好组建农村银行工作，力争村镇银行覆盖所有县（市），新增小额贷款公司和担保公司15家以上。积极培育上市后备资源，支持中小企业在创业板上市，力争新增上市企业1家以上。积极发展证券、保险、典当等行业，支持发展创业（风险）投资基金和产业投资基金。
增强科技创新能力。推进国家技术创新工程试点，着力培育技术创新体系，确保省级和市级企业技术中心达到50家。培育省、市创新型企业8家，申报认定省高新技术企业5家，高新技术总产值突破120亿元。积极申报建设国家轻纺织品和高分子材料质检中心。加快安庆高新技术创业服务中心、博士后工作站和科技创业园建设，推动企业与高等院校、科研机构开展科技合作与共建，鼓励企业技术创新和研发新产品。抓好人才队伍建设。
（六）深入推动文化强市，促进文化发展繁荣
坚持一手抓公益性文化事业，一手抓经营性文化产业，加速推进旅游业，持续建设文化强市。
推进和谐文化建设。扎实推进社会主义核心价值体系建设，深入开展社会公德、职业道德、家庭美德和个人品德教育，努力提高全民文化素质，形成良好的社会风尚。继续深入开展群众性精神文明创建活动，拓展结对创建、城乡共建、区域联建等形式，积极创建省级文明城市和文明县城、文明村镇、文明单位。
繁荣文化事业。巩固文化体制改革成果，加快经营性文化单位转企改制，建立现代企业制度。继续推进文化惠民工程。加强专业剧团建设，大力发展民间剧社。加强文物遗产保护，完成第三次全国文物普查。加强艺术精品创作，促进地方戏曲艺术繁荣和发展。认真组织庆祝建党90周年、纪念辛亥革命100周年等文化活动。加快中国黄梅戏发展基地建设。
发展文化产业。加快推进文化重大工程项目，扩大产业规模，增强文化产业整体实力和竞争力。打造太湖五千年文博园、岳西国际养生文化产业园、宿松黄梅戏文化主题公园、桐城文化博览园等一批特色文化产业园区。加快培育骨干文化企业，积极引进战略投资者，鼓励社会投资创办、领办、合办文化企业。大力发展新型文化业态，加快表演艺术、影视动漫等创意产业发展。加强文化产品、服务和要素市场建设。提升文化市场管理水平，完善属地综合执法。
加快旅游业跨越发展。推进文化和旅游深度融合，实施旅游跨越行动,继续推进百亿旅游项目工程，加快桐城孔城老街、仙龙湖，枞阳浮山，岳西天峡、妙道山、司空山，宿松石莲洞等项目建设。扎实推进天柱山旅游度假区、花亭湖旅游经济聚集区、龙山凤水文化旅游产业园建设。全年创建5A景区1个、4A景区3个。以列入皖南国际旅游文化示范区为契机，加速融入全国全省旅游网络。
（七）着力保障和改善民生，提高群众幸福指数
坚持以民生工程为抓手，切实解决就业、社保、住房等事关群众切身利益的民生问题，着力提高社会管理和公共服务水平。
拓展提升民生工程。今年计划投入40亿元。省定民生工程33项，其中新增一事一议财政奖补试点、农村公路危桥加固改造工程、家电下乡和家电以旧换新等3个项目。市定民生工程9项，其中新增城区人口出生缺陷干预一级预防试点项目。提高农村五保户供养、重度残疾人生活和农村义务教育阶段生均公用经费补助标准，扩大对高校、中职学校家庭经济困难学生资助范围，实施普通高中家庭经济困难学生资助。继续提高新型农村合作医疗受益水平，实现门诊统筹覆盖所有行政村。加快棚户区改造，加快经济适用房、廉租住房和公共租赁房建设进度，扩大住房公积金覆盖面，构建住房保障体系。
高度关注就业和社会保障工作。继续落实更加积极的就业政策，多渠道增加就业岗位。重点做好高校毕业生、农村转移劳动力、城镇就业困难人员、退役士兵就业工作。加强职业技能培训。加快第三批农民工创业园建设。加强社保体系建设，加大对低收入群体帮扶救助，全面推进城乡医疗救助一站式服务，完善临时救助制度。坚持城乡低保动态和分类管理，提升保障水平。继续完善被征地农民养老保险制度，切实抓好新型农村养老保险试点工作，做好政策性农业保险。
加快发展各类社会事业。认真落实教育体制改革各项任务，加快学前教育机构建设，提升义务教育质量，完善高中和职业教育，扩大涉农中等职业教育免费范围，提升高等教育服务地方发展水平，鼓励发展民办教育。扎实推进中小学校舍安全工程。建成黄梅戏学校新校区。支持部队和后备力量建设，巩固发展全省双拥模范城。切实稳定低生育水平，大力提高出生人口素质，遏制出生人口性别比偏高势头。依法开展第八届村委会换届选举。全面启动“六五”普法。扎实推进全国残疾人社会保障和服务体系建设试点。加强宗教事务管理，做好民族工作。加快市立医院、第一人民医院和中医院新区建设。
切实维护社会和谐稳定。加强和改进新形势下群众工作，深入推进社会矛盾化解、社会管理创新、公正廉洁执法，确保社会大局和谐稳定。完善信访工作机制，狠抓社会治安综合治理，强化安全生产监管，努力防范各类重特大事故发生。创新城市社区管理体制和服务体系，健全社会舆情汇集和分析机制，建立重大项目和政策的社会稳定风险评估机制，从源头上预防和减少矛盾。健全政府应急管理机制，提高保障公共安全和处置突发事件的能力。
（八）加强政府自身建设，打造人民满意政府
开创“十二五”发展新局面，对政府自身建设提出了新的更高要求。我们要勇于担当，永不懈怠，不断建设人民满意的政府。
坚持依法行政。贯彻国务院关于进一步加强法治政府建设的意见，切实提高运用法律手段解决矛盾和问题的能力。自觉接受人大及其常委会的法律监督和人民政协的民主监督,认真接受社会公众、新闻媒体和舆论监督。完善重大行政决策机制，推进决策科学化民主化法治化。加强规范性文件清理。加强行政执法队伍建设。深化行政审批制度改革，完善行政审批“两集中、两到位”，推进流程再造，提升机关效能。完善政务目标考核管理，积极争先进位。推进重点领域的政府信息公开。加强改进投诉受理、政风行风评议和政风热线工作。
坚持执政为民。牢固树立宗旨意识和群众观点，始终把群众利益作为想问题、作决策、干工作的出发点和落脚点。坚持思想上尊重群众，感情上贴近群众，工作上依靠群众，从群众中汲取智慧和力量，始终与人民群众同呼吸、共命运、心连心。涉及群众利益的重大决策,广泛征求意见，充分考虑群众诉求和承受能力。积极运用网络、手机信息系统等通讯手段，拓宽联系群众渠道。完善热线电话、网民留言办理、信访绿色邮政、民意调查等制度。
坚持廉洁从政。落实党风廉政建设责任制，推进惩治和预防腐败体系建设，加大教育、监督、改革和制度创新。加强行政监察，强化重点财政专项资金和重大项目审计监督，健全招标采购监管机制，开展工程建设、国土资源等领域突出问题专项治理。大力开展监督检查，严肃查处违法违纪行为。深入开展纠风治乱，切实纠正损害群众利益的不正之风。
各位代表！“十二五”发展的蓝图已经绘就。让我们高举中国特色社会主义伟大旗帜，在中共安庆市委的坚强领导下，万众一心，奋发图强，为实现“十二五”发展的良好开局，作出新的更大贡献！
